# Socially Disadvantaged Community Structures and Conditions Negatively Influence Risky Sexual Behavior in Adolescents and Young Adults: A Systematic Review

**DOI:** 10.3389/ijph.2022.1604488

**Published:** 2022-11-07

**Authors:** Sung-Heui Bae, Jieun Jeong, Youngran Yang

**Affiliations:** ^1^ College of Nursing, Graduate Program in System Health Science and Engineering, Ewha Womans University, Seoul, South Korea; ^2^ Graduate School of Clinical and Public Health Convergence, Ewha Womans University, Seoul, South Korea; ^3^ College of Nursing, Research Institute of Nursing Science, Jeonbuk National University, Jeonju, South Korea

**Keywords:** systematic review, risky sexual behavior, adolescent, community structure, community condition

## Abstract

**Objectives:** This review aims to examine the association between community-level factors, namely, community structure and condition, and risky sexual behavior (RSB) including early sexual debut, having multiple sex partners, and unprotected sex, in adolescents and young adults.

**Methods:** In total, 17 observational studies were identified for review using the Preferred Reporting Items for Systematic Reviews and Meta-Analyses (PRISMA) guidelines. Among the 11,216 identified articles, excluded articles comprised 8,361 duplicates, 2,855 articles by title screening, 893 by abstract screening, and 667 by full-text screening. Finally, eight additional articles were added by manual search.

**Results:** The community structural factors included social disadvantage, economic, employment, education status, racial or ethnic composition, residential stability, and physical environment. The current review found that social disadvantage (six studies) and economic status (10 studies) were most frequently examined. Particularly, higher levels of social disadvantage were associated with higher rates of early sexual initiation, inconsistent condom use, and multiple sexual partners.

**Conclusion:** This study highlights that community structure and conditions in terms of social disadvantages should be addressed to prevent RSB in the young population.

## Introduction

Recent global population data reveal a significant increase in the proportion of young people below the age of 25 years, accounting for 42% (>3 billion) of the global population (World Health Organization [1]. Notably, populations of individuals aged 10–24 years were more prevalent in the least developed countries (31.7%) in contrast to developed countries (16.6%) (United Nations Population Fund [2]. Approximately 70% of both males and females reported adolescents are not psychologically and emotionally mature enough to deal with the negative health consequences of sexual behavior. Therefore, RSB by adolescents and young adults may lead to human immunodeficiency virus (HIV) infections, sexually transmitted infections (STI), unintended pregnancies requiring abortion, and legal conflicts [3]. In 2019, 460,000 young people (aged 10–24 years) were newly infected with HIV worldwide [4]. In the United States, young people (aged between 13 and 24 years) accounted for 21% of all new HIV diagnoses in 2018 and 50% of the 20 million new STIs reported annually. Furthermore, approximately 180,000 babies were born to teenage girls in 2018 (Centers for Disease Control and Prevention [5]. Thus, adolescent sexual and reproductive health is an important public health issue that requires a consistent and innovative approach.

Karvonen and Rimpelä [6] described community structure and conditions as strong determinants of adolescent health behavior. The social disorganization theory states that structurally disadvantaged neighborhoods, characterized by lower socio-economic status, racial and ethnic heterogeneity, and residential stability, are most likely associated with a higher incidence of problematic behaviors than advantaged ones [7, 8]. Furthermore, lower community education and employment status can negatively influence adolescent health outcomes [9]. Neighborhood disadvantage describes the percentage of single households with children below 18 years and households receiving public assistance. Moreover, neighborhood disadvantage influences the high level of inconsistent condom use among high school students [10]. Particularly, higher poverty rates in the community are associated with early sexual debut (<16 years of age [11–13]) and having multiple sexual partners among adolescents and young adults [14].

Understanding how the community influences disparities in sexual health among adolescents is essential to developing effective HIV, STI, and pregnancy prevention programs that function beyond the micro-level, considering individual-, family-, peer-, and school-level characteristics. The ecological system theory states that different types of environments can affect human development [15]. Major aspects of people’s lives occur within society. Thus, the organizational structure and processes within a society can foster personal changes and significantly impact people’s health and behaviors [16, 17]. Health research underscoring the roles of the community and its structure in relation to adolescent sexual behavior is severely limited. Furthermore, systematic reviews or meta-analyses on factors contributing to adolescent RSBs have focused largely on individual or family-related factors such as alcohol consumption [18], media exposure [19], parental monitoring [20], and parent–adolescent sexual communication [21]. However, Decker’s review focused on the relationship between neighborhood characteristics and the reproductive health outcomes of adolescents including adolescent pregnancy, contraceptive use, and teen birth rate [22]. In contrast, this review focused on RSB with the primary purpose of preventing HIV and STI. Therefore, this review aimed to examine the association between community-level factors, particularly community structure and condition, as well as RSB in adolescents and young adults.

## Methods

### Design

The Preferred Reporting Items for Systematic Reviews and Meta-Analyses (PRISMA) guidelines [23] were followed throughout the review process. This review applied a five-step approach [24] comprising problem formulation, literature search, data evaluation, data analysis, and presentation. The search strategy, search selection, quality appraisal, data extraction, and synthesis are presented in this section. Since the study did not involve any human participants, ethical approval was waived.

### Search Strategy

In August 2020, a literature search was conducted across eight electronic bibliographic databases, namely, Cumulative Index to Nursing and Allied Health Literature (CINAHL), Cochrane Library, Elton B. Stephens Company (EBSCO), PubMed, PsycINFO, Web of Science, DataBase Periodical Information Academic (DBpia, Korea), and Research Information Sharing Service (RISS, Korea). Particularly, a combination of keywords was used to search through the title and abstract fields to identify relevant articles published between January 2000 and July 2020. The keywords used were (factor(s) AND sexual behavior) OR (risky sexual behavior) AND (adolescent(s) OR young adult(s)). Thereafter, full-text versions of the retrieved articles were screened based on the pre-specified inclusion and exclusion criteria.

### Inclusion and Exclusion Criteria

The criteria for inclusion comprised studies that were: 1) reporting original research and published in peer-reviewed journals; 2) original research articles with a quantitative approach and non-experimental; 3) either published in English or Korean; 4) reporting on adolescents aged 10–19 years (adolescents were classified as either early: 10–14 years, middle: 15–17 years, and late: 18–19-year [25]) and unmarried young adults aged 20–25 years; and 5) examining the association between community-related factors and RSB among adolescents and young adults. For this study, RSB was defined as early sexual debut, multiple partners, and unprotected sex (i.e., inconsistent condom use). Depending on the study, individual categories or a combination were used as the outcome variable (i.e., RSB). For example, to measure RSB, studies could include varying time frames of recall (e.g., last intercourse, last 3 months, and past year). Studies have provided inconsistent descriptions of early sexual debut, which varies from 11–17 to 11–14 years [26]; 14–16 years [27]; ≤13 years [28, 29]; <14 years [30]; <15 years [31, 32]; and <16 years [33]. This review used Hofmann’s classification of adolescents [25, 34] to include studies that examine sexual debut among individuals below the age of 16 years or the experience of sexual intercourse in primary or middle school. Moreover, multiple sexual partners indicated studies presented with estimated values of the average of sexual partners or, one or more sexual partners in contrast to no sexual experience. Finally, studies that had incomplete information on the study participants and presented only the average age were excluded.

The exclusion criteria involved studies that: 1) focused on sexual minority youth; 2) comprised study participants belonging to a specific population, such as juvenile (arrested) youth, homeless, refugees, individuals living in slums, or military youth; 3) consisted of study participants who were pregnant or had given birth; 4) comprised study participants that had medical problems, such as HIV/AIDS, STIs, or other mental health problems, such as depression, bipolar disorder, schizophrenia, conduct problems, or substance use problems except for alcohol; 5) underscored sexual experience that does not emerge from a consensual relationship, such as sexual violence, abuse, or transactional sex; and 6) the community variable only consisted of residential areas, such as urban, suburban, or rural.

The outcome (i.e., unprotected sex) only focused on inconsistent condom use. Thus, studies examining contraceptive methods, such as pills, contraceptive implants, injections, or intrauterine devices (IUD) were excluded.

Generally, community variables can be divided into two domains: 1) structural factors and conditions of the community, and 2) social processes and mechanisms within the community [22]. Structural factors and conditions define social disadvantage, employment status, education status, household composition, racial or ethnic composition, residential stability, and the physical environment of the community. The social processes and mechanisms within the community describe informal social control, community bonding, mutual trust, community resources, community disorder, community safety, and community norms. The current systematic review of the literature focuses on structural factors and conditions in the community.

### Study Screening

**FIGURE 1:**
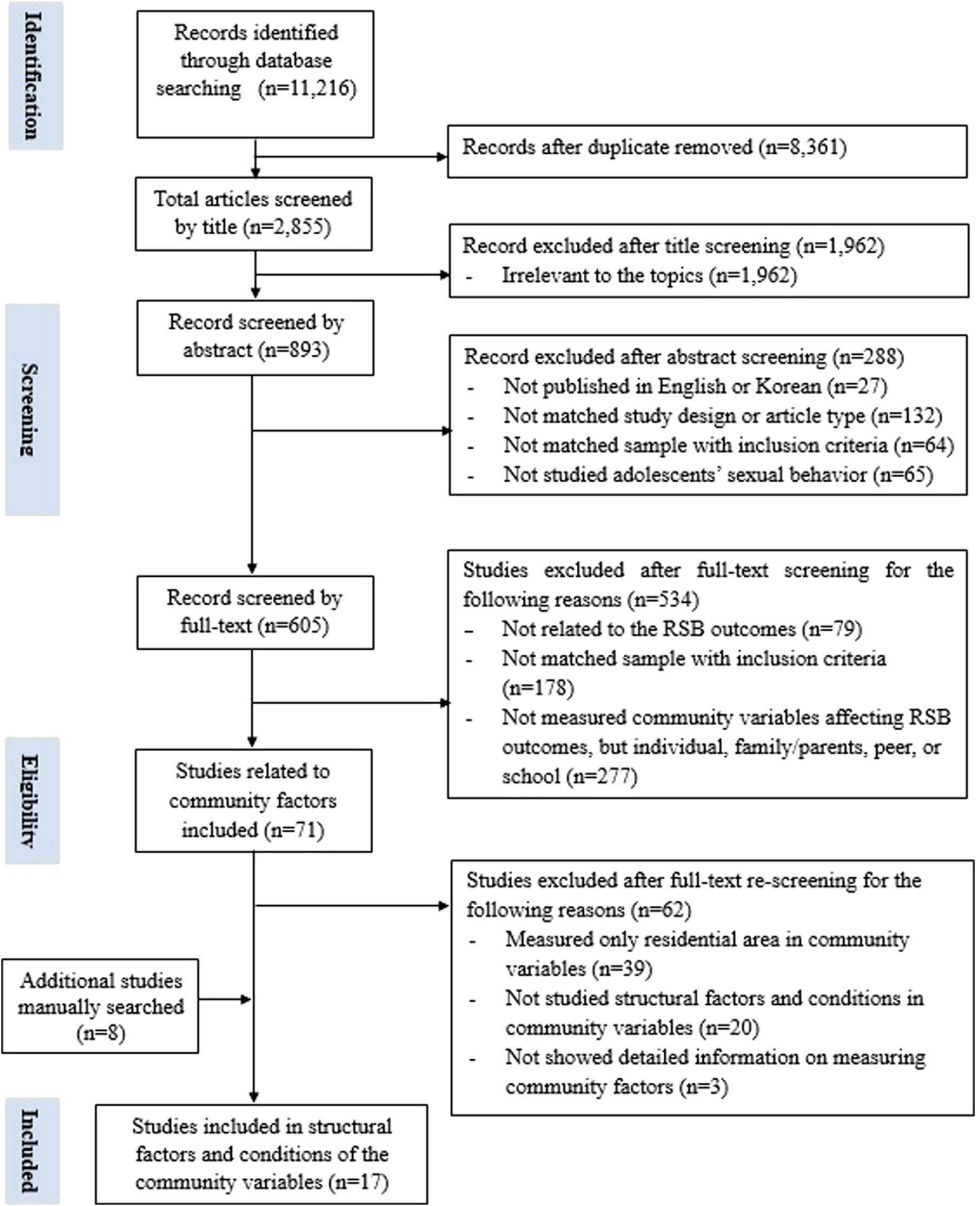
Flowchart of systematic review (Worldwide, 2001-2020).

Figure 1 presents the study selection process using the PRISMA guidelines [23]. Among the 11,216 identified articles, 8,361 duplicates were removed and 1,962 articles were excluded after title screening. The remaining 893 articles were screened based on their abstracts and 288 were excluded.

Following the abstract screening, 79 full-text articles were screened and studies not related to the RSB outcome were excluded. Furthermore, 178 articles were excluded if discrepancies were found with the study samples regarding the inclusion criteria including age, homosexual or bisexual samples, marital status, and members of specific community subgroups (i.e., military youth, adolescent youth, or individuals living in slums). The remaining 348 articles were categorized based on their individual, family or parent, peer, school, and community factors before being reviewed. Thereafter, 277 articles were excluded because the independent variables were not related to the community factors. In the second full-text screening, 71 articles exploring community variables were reviewed thoroughly. Consequently, 39 articles that examined the association between residential area and RSB were excluded. Moreover, 23 articles were excluded because they either did not examine the structural and conditional factors of the community (*n* = 20) or lacked detailed assessments of the community factors (*n* = 3).

Additionally, eight eligible articles were searched and added to the reference list [22]. In total, 17 articles were analyzed. During the screening of the final selected studies, all researchers independently reviewed the title, abstract, and full texts. Any discrepancies or disagreements concerning a study were resolved through discussion and reaching consensus regarding its possible inclusion.

### Quality Appraisal

To ensure the quality and validity of the included articles, all 17 articles were subjected to a quality appraisal *via* a nine-point assessment scale [35]. The original scale was developed for a meta-analysis [36] and was thus revised for the current study. The revised scale used nine items addressing different questions, namely, defined sample, representative sample, inclusion of controls in the analysis, predictors measured, completion rate, demographic information, definition of RSB, details of RSB, and publication status (in a peer-reviewed journal or book). For each criterion, articles received a score of 0 (no) or 1 (yes) which were summed to provide the highest score of 9. According to the adapted tool [36], the categorizations used for the studies were low (score <2), moderate (3–5), or high quality (>6). Consequently, all 17 articles were rated as high-quality articles (Table 1).

**TABLE 1:** Study Quality Scoring for Each Study Included in the analysis (Worldwide, 2001–2020).

Study	Defined sample	Representative sample	Controls in analysis	Predictors measured	Competition rate	Demographic Info	Definition provided	RSB details	Published study	Total/9	Classification (H/M/L)
[10]	1	1	1	1	1	1	1	0	1	8	High
[43]	1	1	1	1	0	1	1	0	1	7	High
[51]	1	0	1	1	1	1	1	1	1	8	High
[11]	1	1	1	1	1	1	1	0	1	8	High
[12]	1	1	1	1	1	1	1	0	1	8	High
[44]	1	1	1	1	1	1	1	0	1	8	High
[48]	1	1	1	1	0	1	1	0	1	7	High
[26]	1	1	1	1	0	1	1	1	1	8	High
[46]	1	1	1	1	1	1	1	1	1	9	High
[13]	1	1	1	1	0	1	1	1	1	8	High
[49]	1	1	1	1	1	1	1	0	1	8	High
[42]	1	1	1	1	0	1	0	0	1	6	High
[14]	1	1	1	1	0	1	1	1	1	8	High
[50]	1	1	1	1	0	1	1	0	1	7	High
[47]	1	1	1	1	1	0	1	0	1	7	High
[45]	1	1	1	1	0	1	1	1	1	8	High
[33]	1	1	1	1	0	1	1	0	1	7	High
Total (N = 17)											7.65

Criteria	Y/N	N (%)	Criteria	Y/N	N (%)	Criteria	Y/N	N (%)
Defined Sample	Yes	17 (100)	Predictors Measured	Yes	17 (100)	Definition Provided	Yes	16 (94.12)
	No	0 (0)		No	0 (0)		No	1 (5.88)
Representativeness	Yes	16 (94.12)	Completion Rate	Yes	8 (47.06)	RSB Details	Yes	6 (35.29)
	No	1 (5.88)		No	9 (52.94)		No	11 (64.71)
Controls in Analysis	Yes	17 (100)	Demo-graphic Info	Yes	16 (94.12)	Published Study	Yes	17 (100)
	No	0 (0)		No	1 (5.88)		No	0 (0)

Criteria for assessing study quality for all studies included in the study		
Title		
FirstAuthor/Year		
**Criterion**	**Description**	**Scoring**
1. Defined Sample	Does the study have a defined sample based on the following elements?	Yes = 1 or No = 0
	▪ defined eligibility and exclusion criteria	
	▪ age range/cutoffs age range	
	▪ an adequate description of the recruitment process	
	The study must meet at least 2 of the above elements to receive a score of 1	
2. Representative Sample	Is the study sample representative of the specific population that it draws from? If representativeness is unspecified, score as 0	Yes = 1 or No = 0
3. Controls in Analysis	Is the sample weighted or controlled for factors such as gender and age? Does the study include a regression analysis to take into account the effect of moderating variables?	Yes = 1 or No = 0
4. Predictors Measured	Does the study measure and report findings on at least one predictor other than gender?	Yes = 1 or No = 0
5. Completion Rate	Does the study report a completion rate?	Yes = 1 or No = 0
6. Demographic Info	Does the study measure and report findings on at least one predictor other than gender?	Yes = 1 or No = 0
7. Definition Provided	Is risky sexual behavior clearly defined? The study must include clear information at least 1 of the following in order to receive a score of 1:	Yes = 1 or No = 0
	▪ Risks: STDs, HIV, unintended pregnancy, abortion	
	▪ Sexual activity: early debut, unprotected	
	▪ Partner: irregular, incentive-driven, multiple	
8. Risky Sexual Behavior details	Does the study provide details (excluding gender and ethnicity) on risky sexual behavior? For example, are prevalence rates provided for sub-groups or specific risky sexual behavior details? At least three details need to be reported to receive a score of 1. Examples include:	Yes = 1 or No = 0
	▪ Risky Sexual Behavior broken down by age groups, family types, relationship status, etc.	
	▪ Detailed description of RSB	
9. Publication Status	Is the study published (peer-reviewed journals, book chapters)?	Yes = 1 or No = 0
Classification	The classification system used identified studies of low (<2), moderate (3–5), or high (>6) quality	Score ( )
		High/Mod/Low

Legend: The individual quality score items are summed to generate a total score for each study.

Total scores range from 0 to 9. Articles were given a score of 0 (“No”) or 1 (“Yes”) for each criterion and summed to give a total score out of 9.

The classification system used identified studies of low (<2), moderate (3–5), or high quality (>6). The average study quality score was 7.65.

That of all studies (100%) fell in the high-quality range. For additional information on Quality scoring was referenced in the study of [36].

### Data Extraction and Synthesis

For this review, information was extracted from the selected articles and tabulated to compile the extracted data (Table 2), namely, author(s), year of publication, country of study, study design, study setting, source of primary data, sample size or demographic information, theoretical framework, outcome variables, community-related variables, data analysis, and results. Furthermore, the relationship between community factors and RSB in adolescents and young adults was further categorized into a significant or non-significant relationship.

**TABLE 2:** Characteristics of included articles (Worldwide, 2001-2020).

	Author (year), country	Study type, analysis, setting, sample	Primary data source	Theoretical framework	Outcome variables	Community related variables	Findings significant relationships with RSB (*p* ≤ 0.05)
[1]	[10], United States	Longitudinal; Hierarchical linear model; School/community (visiting) N = 681 Age range= Grade 9–12 (Mean 14.8 years) F (51%), M (49%)	Flint Adolescent Study, 1994–2004	Social disorganization theory (Clifford Shaw and Henry McKay, 1942)	Inconsistent condom use at last sex: ranging 0–4, always to almost never	Neighborhood Economic Disadvantage (Alpha co -efficient =0 .89): % of poverty; Single-headed households with children under the age of 18; Household receiving public assistance; Households earning less than $15,000; % of residents without a high school diploma; % of area unemployment ^a^ Developed by this study	Significant relationship between neighborhood disadvantage and initial condom use (B = −0.10, SE = 0.04); Significant relationship between single-headed households with children under the age of 18 and initial condom use (B = −0.10, SE = 0.003); Significant relationship between households earning less than $15,000(B = 0.004, SE = 0.002); and households receiving public assistance and initial condom use (B = −0.006, SE = 0.003); NS relationship between proportion of households less than a high school degree or with varying unemployment rates and initial condom use; NS relationship between neighborhood economic disadvantage and change in condom use over time
[2]	[43], United States	Longitudinal; Proportional hazard regression and logistic regression; Home (visiting); N = 1,111Age range = 18–22 (First sex mean age = 16.5)F (51%), M (49%)	1. NSC, 1976–19872.1980 Census	N/A	Number of sex partners before 1 year: open-ended	Neighborhood Disadvantage Index (Alpha Coefficient =0 .90): Poverty rate; % of families received public assistance; % of families earned less than $30,000; Male joblessness rate (i.e., % of working-age men: either unemployed or not in the labor force); % of persons aged 25 and older without a college education; % of workers who were not in managerial or professional occupations	Significant relationship between neighborhood disadvantage and number of sex partners (B = 0.016, SE = 0.012)
[3]	[51], United States	Cross-sectional; Logistic regression; Home (online); N = 921Age range = 12–16 (Mean 16.2 years) F (49%), M (51%)	Media exposure and adolescent sexual behavior survey (A three waves longitudinal), Wave 1, 2005	N/A	1. Lifetime number of partners: open-ended; 2. Condom use: ranging 0–5, never to always	Physical availability of FPC: Distance to nearest FPC (miles from each adolescent’s home to nearest FPC); Travel time to nearest FPC (density of FPCs within 1-mile and 3-mile radii of home)	NS relationship between physical availability of FPC and number of sexual partners; NS relationship between physical availability of FPC and condom use
[4]	[11], United States	Longitudinal; Logistic regression; Multimethod (Home, community); N = 915Age range = 11–16 (Mean 13.3 years) F (52.4%), M (47.6%)	1. PHDCN, Wave 1,1994–19972. 1990 Census	N/A	Age at sexual intercourse: open-ended	Concentrated poverty; Residential stability & % of housing occupied by owners; Immigrant concentration (combined percentage Latino and percentage foreign born)	Significant relationship between concentrated poverty and age at sexual intercourse (B = 0.552, SE = 0.132); NS relationship between residential stability and percentage of housing occupied by owners age at sexual intercourse; NS relationship between immigrant concentration age at sexual intercourse
[5]	[12], United States	Longitudinal; Multilevel discrete-time logit model; Multimethod (Home, community); N = 907Age range = 11–16 (Mean 13.3 years) F (52.4%), M(47.6%)	1. PHDCN, Wave 1,1994–19972. 1990 Census	N/A	Age at sexual intercourse: open-ended	Concentrated poverty; residential stability and % of housing occupied by owners; Immigrant concentration (combined percentage Latino and percentage foreign born)	Significant relationship between age at sexual intercourse and concentrated poverty (B = 0.454, SE= 0.180); NS relationship between age at sexual intercourse and residential stability; NS relationship between age at sexual intercourse and immigrant concentration.
[6]	[44], United States	Longitudinal; Poisson regression; Multimethod; N = 6,985Age range = 12–16F (48%), M (52%)	1. NLYS97,1997–2002 2. 2000 Census	N/A	Sex partners (12 months): one or one more partner, open-ended	Neighborhood Disadvantage (Coefficient alpha= 0.82): % of residents below the official poverty threshold; % of residents (16+) who are unemployed; % of households headed by a female with children less than eighteen living in the household	Significant relationship between neighborhood disadvantage and number of sexual partners (B = 0.01, *p* <0 .01)
[7]	[48], South Africa	Longitudinal; Logistic regression; Home (visiting); N = 2,992 Age range = 14–22F (55%), M (45%)	Transitions to Adulthood in the Context of AIDS in South Africa, 2001	N/A	1. Condom use (12 months): 0= If a condom had not been used with one or more partners, 1= If a condom had been used at last sex for all partners	Community education norms (% of young people enrolled at any level of school; % of people aged 20 and older who graduated from secondary school); Norms of employment (% of adolescents performing wage labor in a community; the wages they earn per week)	(Male) Significant relationship between proportion of primary or secondary school and condom use (OR = 0.04, *p* = 0.049); Significant relationship between proportion of graduated from secondary school and condom use (OR = 0.01, *p* = 0.031); Significant relationship between current working status and condom use (OR = 0.01, *p* = 0.019); NS relationship between average earnings per week and condom use; (Female) Significant relationship between average earnings per week and condom use (OR = 1.59, *p* = 0.003); NS relationship between proportion of primary or secondary school/proportion of graduated from secondary school/current working status and condom use
[8]	[26], United States	Longitudinal; Logistic regression; Home (online); N = 2,649 Age range = 11–14F (56%), M (45%)	1. Add health, Waves 1–2,1994–1996 2.1990 census	N/A	early sexual initiation: yes/no	Neighborhood poverty concentration: ≤5%, >5%–10%, >10%–20%, >20%^a^ % of families in the adolescents’ census tract of residence living below the federal poverty level	NS relationship between neighbor hood poverty concentration (per 10% increase) and early sexual initiation.
[9]	[46], South Africa	Longitudinal; Probit regression; Home; N = 2,993, African (1,410); Colored (1,583) Age range = 17–22 AF (54%), AM(46%) CF(51%), CM(49%)	1. CAPS, 2002–20052. 2001 Census	N/A	1. Multiple sexual partners in past year: open-ended 2. Condom use at last sex: ranging 1–4, always to rarely	Community poverty rate: The community poverty rates are computed as the proportion of households living below the poverty line (R9, 600 per household per year) in 2001	(Male) Significant relationship between living in poorer community and condom use (Marginal effect = −0.459, SE = 0.24); NS relationship between community poverty and multiple partners. (Female) NS relationship between community poverty and multiple partners/condom use

[10]	[13], South Africa	Longitudinal; Probit regression; Home; N = 2,993 Age range = 14–22 (Mean 17.8 years)F (52%), M (48%)	1. CAPS, 2002–20052.2001 Census	N/A	1. Sexual experience: 0= no, 1= yes^a^ Whether youth had sexual debut between 2002 and 20052. Multiple sexual partners: 0= 0 or 1 1 = more than 1 3. Condom use: 0= used condom, 1 = nonuse condom	Community poverty rate: The community poverty rates are computed as the proportion of households living below the poverty line (R9, 600 per household per year) in 2001	(Male) Significant relationship between community poverty and earlier sexual debut (Marginal effect = 0.462, SE = 0.232); NS relationship between community poverty and Multiple partners/Condom use; (Female) NS relationship between community poverty and early sexual debut/multiple partners/condom use
[11]	[49], United States	Cross-sectional; Multinominal regression; Public school; N = 2,150 Grade = 10–12 gradeF (48.4%), M(51.6%)	1. E2S-YES, 2012	N/A	1. Inconsistent condom use or birth control 2. Two or more sexual partners in the past year: 2 items with open-ended	Transitions and mobility (4 items, alpha coefficient = 0.51): How many times have you changed homes since kindergarten?	Significant relationship between transitions and mobility and RSB (AOR = 1.73, 95% CI = 1.38–2.18)
			2. CTC				
[12]	[42], United States	Cross-sectional; Logistic regression; Home (online); N = 1,092 Age range = 15–19 M (100%)	1. NSFG, Cycle 4, 2000–20022.2000 Census	N/A	1. Partnering: 3 more sexual partners in lifetime (yes/no) 2. Contraception: used condom at first/last intercourse (yes/no)	Neighborhood disadvantage scale (Coefficient alpha= 0.74, ranging 0–5): % of population with 1999 income below federal poverty level (mean); % of population aged 18–24 years with no high school diploma or equivalent (mean); % of men unemployed (mean); % of family households with own children younger than 18 years with female householder, no husband present (mean); % of 2000 population aged ≥ 5 years not in same house as in 1995 (mean)	Significant relationship between neighborhood disadvantage and multiple partners (OR = 1.23, *p* <0 .01); NS relationship between neighborhood disadvantage and condom use
[13]	[14], South Africa	Longitudinal; Logistic regression; Home (visiting); Wave 1 (*n* = 4,704) Wave 2 (*n* = 1,368) Wave 3 (*n* = 3,426) Wave 4 (*n* = 3,291) Wave 5 (*n* = 2,823) Age range = 14–22 (Mean 17.8 years)F (55%), M (45%)	CAPS, Wave 1–4, 2002–2009	Ecological framework [15]	Multiple sexual partners before 12 months: Yes (2 or more), No (1 person)	Community (proportion of HH): HH unemployed (mean %); HH headed by females (mean %); HH in informal dwelling (mean %); HH Individuals Black Africans (mean %); HH below poverty line (mean %)Additional community level attributes were defined by sub-place on the mean annual incomes and schooling years	(Male) NS relationship between % of HH unemployed/% of HH headed by females and MSP; Significant relationship between % of HH in informal dwelling and MSP (LF = −0.42); Significant relationship between % of individuals African residents and MSP (LF = 1.37); NS relationship between mean annual house incomes and MSP; Significant relationship between % of HH below poverty line and MSP (LF = 0.69); (Female) NS relationship between % of HH unemployed and MSP; Significant relationship between % of HH headed by females and MSP (LF = 0.27); NS relationship between % of HH in informal dwelling and MSP; Significant relationship between % of individuals African residents and MSP (LF = −1.22); NS relationship between mean annual house incomes and MSP; Significant relationship between % of HH below poverty line and MSP (LF = 0.67)
[14]	[50], United States	Longitudinal; Hierarchical multilevel regression; Home (multimethod) N = 4,179 Age range = 11.01 at Wave 1, 16.10 at Wave 3 F (50.8%), M(49.2%)	Healthy passage (A multi-site longitudinal investigation of adolescent health behaviors), Wave 1,32004, 2011	N/A	1. Sexual initiation: age of first vaginal sex (ranging 1–9, 10 years old to 18 years or older) 2. Number of sex partner: open-ended	1. Concentrated poverty (US census, 2000): Economic disadvantage (Gardner et al., 2012, 5 items alpha coefficient = 0.92) 2. Neighborhood decay (Peterson et al., 2007): Commercial Decay (15 items, alpha coefficient =0.89); Residential Decay (12 items, alpha coefficient =0.74)	NS relationship between concentrated poverty and age of first vaginal sex/number of sex partners; NS relationship between commercial decay and age of first vaginal sex/number of sex partners; Significant relationship between residential decay and age of first vaginal sex (B = −0.08, SE= 0.04); NS relationship between residential decay and Number of sex partners
[15]	[47], Canada	Longitudinal; Multiple regression; HomeN = 2,596 Age range = 10–19^a^ Measured sexual activity age at 16–17 or 18–19 F (51%), M (49%)	1. NLSCY, 1994–1995, 2002–20032.2001 Canadian Census	Ecological model [15] and Developmental model [40]	1. Timing of first consensual sexual intercourse: Having consensual sexual intercourse (yes/no) 2. Age of the first consensual sexual intercourse: open-ended	Neighborhood poverty (Poor or Nonpoor): The DA was the geographic unit used to approximate the neighborhood environment. All DAs with 20% or more residents under Statistics Canada’s low-income cutoff were considered as poor neighborhoods	NS relationship between neighborhood poverty and timing of first consensual sexual intercourse
[16]	[45], United States	Longitudinal; Logistic regression; Home (online); N = 820Age range = 12–19 (Mean 15 years) F (54%), M (47%)	1. TARS, Wave 1,2, 2001–2002 2.2000 US Census	N/A	1. Sexual debut: 1= yes, 0 = no 2. Number of sexual partners: ranging 1–11	Neighborhood Disadvantage (alpha coefficient =0.96)	NS relationship between neighborhood disadvantage and sexual debut/number of sex partners
[17]	[33], United States	Longitudinal; SEM; Home or School (middle, junior and high schools); N = 14,058Age range = 10–22 (Mean 15.14 years) F (52%), M (48%)	1990 US Census	Life Course Theory (=life course perspective [41])	1. Early sex before 16 years: 0 = no, 1 = yes 2. Infrequent condom use (before 12 mo.): ranging 0–4, every time to never use 3. Number of sex partners (before 12 mo.)	Community Socioeconomic Disadvantage index (alpha coefficient = 0.78, ranging 0–4):% of families living in poverty; % of single-parent families; % of adults employed in service occupations; % of unemployed males	Significant relationship between community disadvantage and early sexual engagement (logistic coefficient B = 0.48, SE = 0.12); Significant relationship between community disadvantage and infrequent condom use (logistic coefficient B= −0.31, SE = 0.10); NS relationship between community disadvantage and multiple partners

^a^ Add health = The National Longitudinal Study of Adolescent to adult Health; AF, african females; AM, african males; CAPS, the cape area panel study; CF, colored females; CM, colored males; CTC, communities that care; DA, dissemination area; E2S-YES, Evidence2Success, Youth Experience Survey; FPC, family planning clinics; HH, household; IRR, incidence rate ratio; LF, linear discriminant function coefficient for significant factors at the multivariate mode, MSP, multiple sex partners; NLSCY, the national longitudinal survey of children and youth; NLSY, national longitudinal survey of youth; NSC, the national survey of children; NSFG, the national survey of family and growth; OR, odds ratio; PHDCN, Project in Human Development in Chicago Neighbor-hoods Community Survey; SE, standard error; SEM, multilevel structural equation models; TARS, toledo adolescent relationships study.

## Results

### Study Characteristics

Among the 17 studies reviewed, 12 were conducted in the US, 1 in Canada, and 4 in South Africa. Fourteen studies applied a longitudinal study design, while the remaining three used cross-sectional designs. The sample sizes ranged from 691 to 14,058 participants and most studies included both women and men, except one that focused exclusively on men. To investigate the relationship between community structure or conditions and different RSB outcomes in this population, many studies used large administrative datasets such as the National Longitudinal Study of Adolescent to Adult Health (Add Health) data and the National Longitudinal Survey of Youth (NLSY) in the United States, as well as the Cape Area Panel Study (CAPS) in South Africa among other surveys. Add Health contained a national-level representative sample of over 20,000 adolescents from grades 7 to 12 during the 1994–1995 school year and has been followed for five waves to date with most recent update in 2016–2018. Furthermore, the study was designed to assess adolescent health, focusing on multiple social contexts, including homes, schools, neighborhoods, and peer networks [26]. The NLSY cohort also comprised a nationally representative sample of 8,209 adolescents aged from 12 to 16 years in 1997. The survey examined school progress, labor force behavior, and the transition from school to work in the United States [27]. Furthermore, CAPS was a representative longitudinal study conducted in South Africa involving 4,752 adolescents aged 14–22 years in 2002, and followed up in 2005. CAPS concluded that sexual behavior changes over time. Moreover, CAPS suggests that current behaviors are related to a range of household-level variables that were measured earlier in the life of young adults [13, 37].

To synthesize and interpret the findings, theoretical frameworks were applied. One study utilized the social disorganization theory [38] (for an expanded overview, see [39]), one used the ecological model [15], another study employed the ecological [15] and developmental models [40], and another study utilized the life course perspective (life course theory) [41].

All studies used self-reported surveys to examine the RSBs. Some studies included additional variables for RSB, such as substance or alcohol use during sex or sex with someone who uses these substances. The number of studies for each data analysis technique was eight for logistic regression, two for multilevel model, two for probit regression, two for multilevel discrete-time logit model, one for Poisson regression (multimethod), one for multinominal regression, and one for multilevel structural equation models (SEM).

### Concept and Measurement of Structural Factors and Conditions in the Community

As mentioned above, the current study designated structural and condition-related factors of the community under eight categories. To simplify the concept of community factors, a single term was used for consistency even if the variable despite the different name from the different studies (Table 2). Table 3 summarizes the results of the analysis based on the outcomes.

**TABLE 3:** Community variables by risky sexual behavior, of quantitative studies reviewed (Worldwide, 2001-2020).

Community variable	Studies included	Early sexual initiation			Inconsistent condom use			Multiple sexual partners			Other RSBs		
		+	−	NS	+	−	NS	+	−	NS	+	−	NS
**A. Structural factors and conditions**													
**1. Social disadvantage**													
Greater social disadvantage	[1],[2],[6], [12],[16],[17]	[17]		[16]	[1] IC_Dis-advantage, [17]		[12]	[2],[6],[12]		[16],[17]			
**2. Economic status**													
Increased poverty rate	[1],[4],[5],[8], [9],[10],[13], [14],[15]	[4],[5] [10] M		[8], [10] F, [14],[15]	[1] IC_Income, public assistance, [9] M		[1] CC, [9] F, [10]	[13] Poverty line		[9],[10],[13] Annual income, [14]			
**3. Employment status**													
Higher proportion of wage labor	[7]					[7] F	[7] M						
Increased proportion of unemployment rate	[1],[13]						[1] IC			[13]			
Increase proportion of youth idle	[7]					[7] M	[7] F						
**4. Education status**													
Lower proportion with higher education	[1],[7]					[7] M	[1], [7] F						
**5. Household composition**													
Decreased percentage of married households	[1]				[1] IC								
Increased percentage female-headed households	[13]							[13] M		[13] F			
**6. Racial or ethnic composition**													
Higher proportion Black Africans	[13]							[13]					
Higher proportion Hispanic	[4],[5]			[4],[5]									
**7. Residential stability**													
Low residential stability	[4],[5], [11],[13]			[4],[5]				[13] M		[13] F	[11]		
**8. Physical environment**													
Physical availability of Family Planning Clinics	[3]						[3]			[3]			
Greater neighborhood decay	[14]	[14] Residential		[14] Commercial						[14]			

Note: + = Significant positive, − = Significant negative; NS, nonsignificant; F, females; M, males; CC, changed in condom use; IC, initial condom use.

Other RSBs (Risky Sexual Behavior) included the sum of every sexual behavior measured level.

[11] RSB: Inconsistent condom use or birth control, two or more sexual partners in the past year.

Article numbers in Table 3 are according to article numbers in Table 2.

#### Social Disadvantage

Six studies used a neighborhood disadvantage scale or index, combining more than one indicator. All scales included at least one measure of income (most commonly measuring the poverty rate), percentage of families using public assistance, or percentage of families with or without high incomes. Each scale also included a variety of additional indicators, such as employment, education, household composition, residential stability, and physical environment [33]. Examined the association between community disadvantage and adolescent RSB and found that community disadvantage was associated with early sexual debut and inconsistent condom use but not with multiple sexual partners. In contrast, a study found that neighborhood disadvantage was associated with multiple sexual partners but not with condom use [42]. Two studies reported an association between neighborhood disadvantage and multiple sexual partners [43, 44], while one study described an association between neighborhood disadvantage and inconsistent condom use [10]. Meanwhile, Warner [45] did not find a significant relationship between neighborhood disadvantage and sexual debut or number of sexual partners.

#### Economic Status

Nine studies examined the association between economic status, usually measured according to neighborhood poverty, and RSB in adolescents. Browning [11, 12] found a significant association between concentrated poverty and early sexual debut [11, 12], while Dinkelman [13, 46] reported that living below the poverty line was linked to RSB only in men. One of Dinkelman’s studies also found an association between community poverty rate and condom use but not with multiple partners Dinkelman [46], whereas the other study revealed an association of community poverty rate with early sexual debut but not with multiple sexual partners and condom use [13]. A researcher measured the mean annual house income and household poverty line and reported that living below the poverty line was only associated with multiple sexual partners [14]. Another study found that household income and receiving public assistance were associated with inconsistent condom use [10]. Finally, the three remaining studies did not find significant results between neighborhood poverty and adolescent RSB ([26]; Orihuela 2020 [47]).

#### Employment Status

Three studies examined the association between employment status and RSB in adolescents. Clark [48] found that a higher proportion of wage labor can be linked to greater odds of condom use, but only for females. The study also concluded that a greater proportion of idle youth was associated with decreased odds of condom use by males. The other two studies found no significant associations for unemployment status [10, 14].

#### Education Status

Two studies assessed and correlated community education levels with adolescent RSB outcomes, one of which reported that higher education levels were associated with a decreased likelihood of condom use in men (Clark 2004). The other study revealed a significant association between lower education levels and inconsistent condom use [10].

#### Household Composition

Two studies assessed household composition categorized as married households or female-headed households. A study about married households reported that a significant proportion of married households demonstrated inconsistent condom use [10]. In contrast, another study found that female-headed households had a significant association with multiple sexual partners for females [14].

#### Racial or Ethnic Composition

Three studies assessed the impact of racial or ethnic composition at the community level on RSB in adolescents. All racial and ethnicity categories were defined as per a review of the census data by the researchers or through respondent information in a survey. A study found an association between a higher proportion of individuals from the black African community and multiple sexual partners among South African adolescents and young adults [14]. Furthermore, Browning defined immigrant concentration as a combined percentage of Latino and foreign-born American adolescents and reported no significant association between immigrant concentration and RSB among American adolescents [11, 12].

#### Residential Stability

Residential stability refers to the measure of continuity of residence, such as the percentage of residents living in the same house and housing occupied by owners. This factor could affect the sexual behaviors of adolescents through environmental and emotional safety. Four studies examined residential stability at the community level. One study found that informal dwelling was significantly associated with having multiple sexual partners in males [14], while another study found an association between residential mobility and RSB [49]. The remaining two studies found no significant association between residential stability and early sexual debut [11, 12].

#### Physical Environment

Two studies examined how the physical environment at the community level, which includes geographic accessibility from home to family planning clinics (FPCs), commercial decay (e.g., the number of abandoned commercial buildings), or residential decay (i.e., the number of abandoned residential units), may influence RSB in adolescents. Orihuela [50] reported an association between greater residential decay and early sexual debut but not with multiple sexual partners. However, commercial decay was not significantly related to RSB [50]. While the remaining study found no significant results for geographic accessibility with FPCs [51].

## Discussion

This review examined the relationship between the community’s structural factors and their impacts on the sexual behaviors of adolescents. Consequently, eight concepts were found for community structure, namely, social disadvantage, economic status, employment status, education status, household composition, racial or ethnic composition, residential stability, and physical environment. Furthermore, social disadvantage (*n* = 6) and economic status (*n* = 10) were examined most often while evaluating their impacts on RSB in adolescents and young adults. After controlling the effect of individual- and family-level variables, this study found that the macro-level factors (i.e., community structural factors), were associated with RSB in adolescents, implying that the community influences the behaviors of its adolescent and young adult members.

Social disadvantage was moderately associated with RSB in young people, with most studies emphasizing the importance of social disadvantage in preventing adolescent RSB. Furthermore, this study found that increased social disadvantage exacerbated the risks of early sexual initiation, inconsistent condom use, and multiple sexual partners. Moreover, non-significant relationships were reported by several authors. However, more than half of the relationships (6 out of 10 relationships) between social disadvantage and adolescent RSB were significant with an expected direction.

The existing reviews on this subject also reported similar results, underscoring the impact of higher neighborhood disadvantage on earlier sexual onset [22, 52]. Another review found that social disadvantage affected sexual behaviors and sexual health risk among indigenous Australian adolescents [53]. However, Decker et al. [22] found mixed results for the association between neighborhood disadvantage and contraceptive use. Table 2 presents the size of the effect and the analysis methods with a p-value of 0.05. Three studies [33, 42, 45] found insignificant relationships and did not report exact p-values. Consequently, it was not possible to determine whether the p-values of the insignificant results were close to 0.05 and the coefficients indicate a large impact in the expected direction. Furthermore, the measurements of social disadvantage vary for each study. Thus, the effect size could not be synthesized with a meta-analysis. Future studies ought to determine how substantial a change in social disadvantage should be to ascertain a relevant impact on sexual behavior.

The results regarding the existence of a relationship between economic status and adolescent RSB were inconclusive. Furthermore, this review found that the evidence supporting both significant and non-significant relationships between increased poverty rate and RSB among adolescents was comparable. However, the findings contradict a previous review [22] that described fairly consistent associations between neighborhood poverty and a decreased likelihood of contraceptive use. Previous studies also reported that income inequality at the community level is associated with certain health outcomes, including injuries, general physical symptoms, limiting conditions, mental health, health behaviors, and physical health of adolescents [9]. Furthermore, provincial income inequality was related to certain physical (e.g., injuries and general physical symptoms) and mental health issues in young adults [9]. These studies found moderately supportive evidence for a relationship between community income and health outcomes in adolescents. Thus, this study cannot state conclusively whether higher poverty rates lead to increased RSB in adolescents and young adults. While these variables measure similar items, economic status is usually a simple measure of neighborhood poverty rate while social disadvantage is a more complex measure. Typically, social advantage includes a diverse set of indicators such as percentage of single-parent households, percentage of households without a car, and percentage of non-employed adults under 65 [55].

Few studies have evaluated the roles of employment status (*n* = 4), education status (*n* = 2), and household composition (*n* = 3) in the young population’s adoption of RSB. Notably, these community structures are significantly correlated with inconsistent condom use and multiple sexual partners. Research also found consistent associations between neighborhood education and employment and adolescent reproductive health outcomes [22]. However, a definitive conclusion could not be made because of the small number of studies exploring this association.

Previous researchers have analyzed the relationship between a higher proportion of Black Africans and Hispanics in the community and RSB and found no indication of their effect on early sexual initiation or inconsistent condom use. Only one study [14, 56] reported a significant relationship between a higher proportion of black Africans and multiple sexual partners in South Africa, consistent with Decker et al. [22]. Nevertheless, it remains unclear whether racial or ethnic concentrations within a community affect the behavior of its young population. Consequently, it was difficult to define the extent to which a certain racial and ethnic group dominates a community. Some authors also pointed out the intricacies of analyzing racial segregation in a community [22,57]. According to an analysis of the data from the National Longitudinal Survey of Youth from 1997 to 2007, African American adolescents exhibited higher sexual risk than Caucasian adolescents by age 19. However, the risk in whites increased thereafter [57]. Thus, the effect of racial segregation on sexual behavior may change over time. Additionally, even if segregation may not be associated directly with sexually risky behaviors, it can influence sexual risk through another mechanism, such as sexual network patterns. Thus, these ideas should be considered in future research.

Lastly, there is insufficient evidence to determine the extent of the influence of residential stability and the physical environment of a community on the sexual behaviors of its adolescent and young adult population. Consequently, most studies found non-significant relationships between these community factors and RSB in adolescents.

Neighborhood disadvantage could further limit the availability of social capital [58] and increase vulnerability to HIV/STIs through RSBs in adolescents and young adults [10]. The current findings also emphasize the principal value of community investment to enhance safer sexual behaviors among the young population. Furthermore, the current findings provide information about high-risk groups regarding RSBs in adolescents and young adults. The findings also underscore the need to monitor and develop programs to ensure safer sexual behaviors among the young population. Particularly, improving the robustness of the police for the young population and their families as well as increasing connections in the community of socially disadvantaged populations may reduce this risk [44]. Moreover, community-level interventions, such as community mobilization and female empowerment, have a positive effect on adolescent issues like sexual and reproductive health [59]. Consequently, increasing emphasis has been placed on community engagement and the implementation of community-developed and community-driven programs [53].

To develop and implement relevant programs and policies, it is important to ascertain the magnitude of changes in the community factors that can produce a relevant social outcome at a reasonable cost. Depending on the financial constraints in the health sector, cost-effectiveness has been used widely to evaluate the costs and health impacts of interventions to optimize resource allocation and maximize the target population’s health [60]. Based on the findings of the present study, the costs of community-level interventions or individual or family-level interventions with socially disadvantaged populations can be examined. Furthermore, their social impacts, including sexual behavior outcomes or health outcomes (e.g., STI, HIV infections, or related deaths) could be used for cost-effective analyses. Cost-effective health interventions for HIV testing [60] and gonorrhea vaccination [61] were also examined. The current findings indicate that intervention programs that target adolescents and young adults living in relatively disadvantaged communities need to be developed. Simultaneously, it is necessary to evaluate their cost-effectiveness before large-scale implementation.

Increased emphasis should be placed on improving a single factor, such as the economic or employment status of the community, as well as to target general social disadvantages within the community such as lower income, poverty rate, percentage of families using public assistance, employment, education, household composition, residential stability, and physical environment. This type of approach can potentially reduce the prevalence of RSBs and their adverse health consequences within the community. Notably, improving health outcomes arising from disparities in community conditions and resource gaps will require a substantial amount of time. Thus, the government’s annual health agenda should include adolescent sexual health among its priorities. A multisector approach (e.g., ministries of education, health, welfare, labor, housing, and environment) and interventions involving community collaborations should be employed. Moreover, empowerment activities should be carried out vigorously.

This systematic review comprised several limitations. First, a causal relationship between community structure and adolescent RSB could not be inferred from this study design. Furthermore, some of the included studies were cross-sectional and thus it was not possible to determine a causal relationship. The current study included a variety of community structure factors, heterogeneous variables and measures, and different analytic models. Consequently, it was not possible to conduct a meta-analysis. Moreover, this study intended to include all studies examining the community structure and RSB in adolescents and young adults. However, the search methods might have limited the scope for the inclusion of all published studies.

In conclusion, this review found notable evidence that community structure, especially social disadvantage, was significantly associated with RSB in adolescents and young adults. Social disadvantage is a multifaceted component, including income, poverty rate, and other community structure factors. Therefore, the current study findings can be used to develop and implement prevention and education programs that target the young population, with a particular emphasis on the socially disadvantaged section of the community. The findings also provide a foundation for facilitating the formulation of a tailored health policy that can prevent RSB in this population and promote reproductive health. Future studies need to address the extent of changes in these community factors, which have socially relevant impacts and related costs, to determine cost-effective programs and policies.
